# Physicochemical Properties of Jatropha Oil-Based Polyol Produced by a Two Steps Method

**DOI:** 10.3390/molecules22040551

**Published:** 2017-03-29

**Authors:** Sariah Saalah, Luqman Chuah Abdullah, Min Min Aung, Mek Zah Salleh, Dayang Radiah Awang Biak, Mahiran Basri, Emiliana Rose Jusoh, Suhaini Mamat

**Affiliations:** 1 Chemical Engineering Programme, Faculty of Engineering, Universiti Malaysia Sabah, Jalan UMS, Kota Kinabalu 88400, Sabah, Malaysia; s_sariah@ums.edu.my; 2 Department of Chemical and Environmental Engineering, Faculty of Engineering, Universiti Putra Malaysia, Serdang 43400, Selangor, Malaysia; dradiah@upm.edu.my (D.R.A.B.); suhainim@unikl.edu.my (S.M.); 3 Higher Institution Centre of Excellence Wood and Tropical Fibre (HICoE), Institute of Tropical Forestry and Forest Products, Universiti Putra Malaysia, Serdang 43400, Selangor, Malaysia; minmin_aung@upm.edu.my (M.M.A.); tintahikmah@gmail.com (E.R.J.); 4 Department of Chemistry, Faculty of Science, Universiti Putra Malaysia, Serdang 43400, Selangor, Malaysia; mahiran@upm.edu.my; 5 Radiation Processing Technology Division, Malaysian Nuclear Agency, Kajang 43000, Selangor, Malaysia; mekzah@nuclearmalaysia.gov.my; 6 Universiti Kuala Lumpur, Malaysian Institute of Chemical and Bioengineering Technology (UniKL MICET), Alor Gajah 78000, Melaka, Malaysia

**Keywords:** jatropha oil, polyol, epoxidation, oxirane ring opening, polyurethane

## Abstract

A low cost, abundant, and renewable vegetable oil source has been gaining increasing attention due to its potential to be chemically modified to polyol and thence to become an alternative replacement for the petroleum-based polyol in polyurethane production. In this study, jatropha oil-based polyol (JOL) was synthesised from non-edible jatropha oil by a two steps process, namely epoxidation and oxirane ring opening. In the first step, the effect of the reaction temperature, the molar ratio of the oil double bond to formic acid, and the reaction time on the oxirane oxygen content (OOC) of the epoxidised jatropha oil (EJO) were investigated. It was found that 4.3% OOC could be achieved with a molar ratio of 1:0.6, a reaction temperature of 60 °C, and 4 h of reaction. Consequently, a series of polyols with hydroxyl numbers in the range of 138–217 mgKOH/g were produced by oxirane ring opening of EJOs, and the physicochemical and rheological properties were studied. Both the EJOs and the JOLs are liquid and have a number average molecular weight (*M*_n_) in the range of 834 to 1457 g/mol and 1349 to 2129 g/mol, respectively. The JOLs exhibited Newtonian behaviour, with a low viscosity of 430–970 mPas. Finally, the JOL with a hydroxyl number of 161 mgKOH/g was further used to synthesise aqueous polyurethane dispersion, and the urethane formation was successfully monitored by Fourier Transform Infrared (FTIR).

## 1. Introduction

The continuous depletion of fossil oil has led to the ever increasing price of such oil, which subsequently has contributed to the increasing price of polymer raw materials. There is a significant trend nowadays with regard to utilising products derived from natural molecules, such as vegetable oil, which is due to the lower cost and the fact that the source is renewable. In addition, bio-based products are more environmentally friendly due to possibility of being biodegradable [1]. Vegetable oils such as soybean oil, castor oil, rapeseed oil, rubberseed oil, palm oil, canola oil, and linseed oil are among the most important classes of renewable resources that have been utilised for the preparation of biopolymer products such as alkyd resins [2,3] and polyurethane [1,4,5].

**Figure 1:**
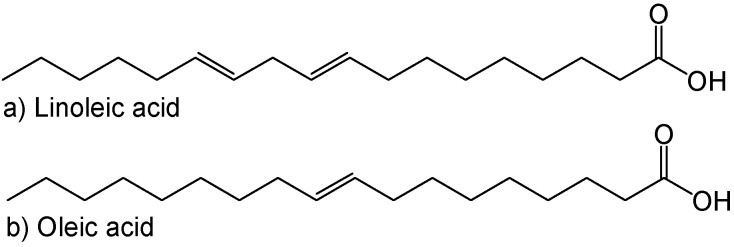
Chemical structure of main fatty acids in jatropha oil.

Currently, polyol derived from jatropha oil appears a promising candidate as a raw material for polyurethane adhesives [6] and coatings [7,8]. Jatropha oil is a non-edible oil, and utilising such an oil does not exploit or harm the market for edible vegetable oil. As shown in Table 1, jatropha oil consists of 78.9% unsaturation percentage in its triglyceride structure, mainly corresponding to oleic and linoleic acid (Figure 1), which provides an interesting platform for chemical modification. The double bond group in the jatropha oil triglyceride molecules can be functionalised to a hydroxyl group by a two‑step method; namely, epoxidation and oxirane ring opening.

Studies of the epoxidation of vegetable oils have become interesting as the products have become important oleochemicals for commercialisation. Despite being used as a starting material to produce polyol, vegetable oils with epoxy groups are now accepted as an alternative to petrochemical based feedstock to be used as plasticizers for polyvinyl chloride, stabilizers, and lubricants [10].

Certain parameters such as the reaction time, the molar ratio of hydrogen peroxide to the oil double bond, and the molar ratio of either formic or acetic acid as the peroxygen carrier to the oil double bond can be manipulated to obtain epoxidised oil with a different oxirane oxygen content (OOC). Higher amounts of peroxygen carrier and hydrogen peroxide as well as higher temperatures will increase the reaction rate, but it is difficult to control the temperature since the reaction is exothermic. The use of a low percent strong acid as a catalyst has been reported [11], but, due to the environmental issue of the waste sulphuric acid, the use of an ion exchanger catalyst has become the preferred approach [12]. In addition, an ion exchanger is preferred because sulphuric acid is reported to be unsuitable for certain oils due to the low yield of epoxide [13]. In some studies, the use of a catalyst is even eliminated, but the more concentrated hydrogen peroxide has to be employed together with a higher loading of formic acid [13,14]. Meyer and co-workers [15] reported that epoxidised jatropha oil (EJO) could achieve an OOC of up to 4.75% at a reaction temperature of 50 °C and 10 h of reaction time. By increasing the reaction temperature to 65 °C, the reaction time can be reduced to 5 h with OOC reduced to 4.3% [15].

Producing polyol from epoxidised vegetable oil involves the opening of the oxirane group to produce a hydroxyl group. A higher OOC value is favourable if a high hydroxyl value of polyol is expected. The hydroxyl value of polyol refers to the average distribution of OH groups present in the triglycerides [16], which will determine the properties of the polyurethane. However, a systematic study of the effect of the epoxidation condition on the properties of polyol derived from jatropha oil is not available.

The aim of this study is to investigate the effect of the molar ratio, reaction temperature, and time on the OOC of EJO. During the epoxidation step, less concentrated hydrogen peroxide will be utilised and the reaction will be performed without using a strong acid catalyst. It is intended to establish the relationship between the hydroxyl value of the polyol and the oxirane oxygen content of the epoxidised oil. Other physico-chemical properties of the EJOs and JOLs, such as the molecular weight and rheology, will be investigated as part of the required information for handling and processing purposes. Furthermore, the potential of producing polyurethane dispersion from the synthesised jatropha oil-based polyol will be investigated.

## 2. Results and Discussion

**Figure 2:**
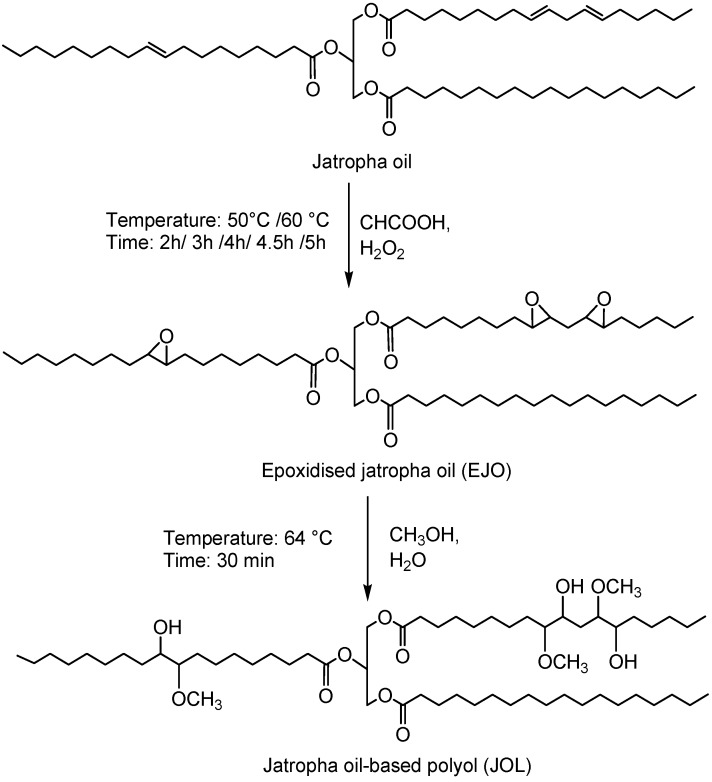
Synthesis of jatropha oil-based polyol via the epoxidation and oxirane ring opening method.

**Figure 3:**
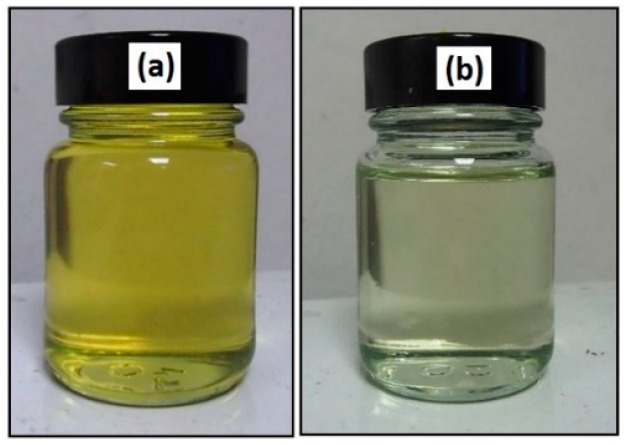
Appearance of jatropha oil‑based polyol (**b**); compared with the starting jatropha oil (**a**).

Jatropha oil-based polyols were produced by the epoxidation and oxirane ring opening route, as shown in Figure 2. In the jatropha oil triglyceride, the oil double bond in the linoleic and oleic fatty acid was transformed to epoxy, which was then opened to incorporate a hydroxyl and methoxy group. The resulting jatropha oil-based polyol contained a secondary hydroxyl group. The formation of epoxidised jatropha oil and polyol were confirmed by FTIR analysis. On the other hand, the epoxy groups in the epoxidised oil were reported as oxirane oxygen content (OOC), while the amount of hydroxyl groups formed in the polyol was reported as the OH number. Photographs of the jatropha oil and the resulting polyol are shown in Figure 3. Jatropha oil is a golden yellow oil, while the polyol is slightly yellow.

### 2.1. FTIR Analysis of JO, EJO, and JOL

**Figure 4:**
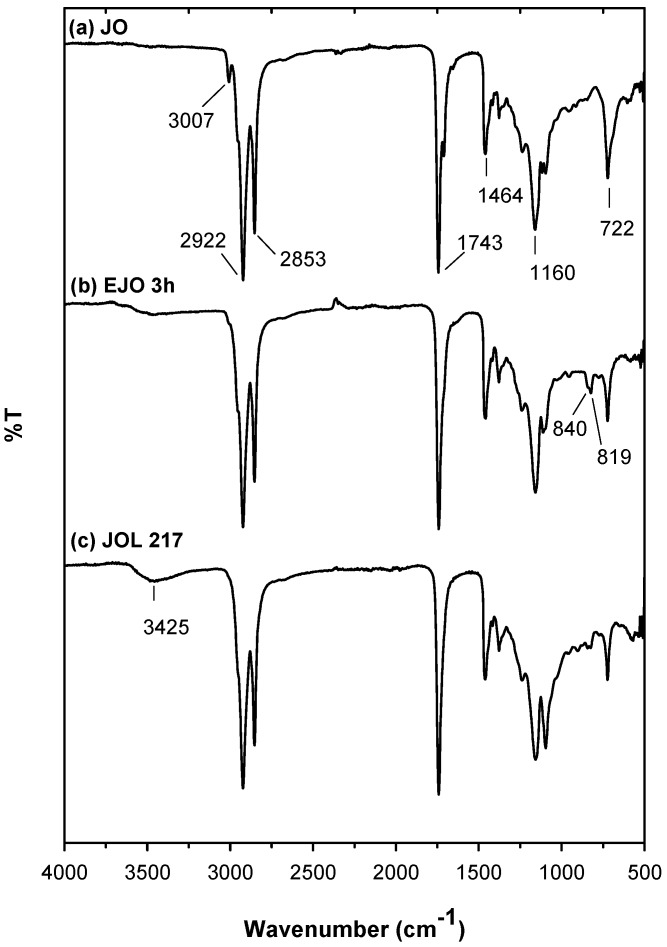
(Fourier Transform Infrared) FTIR spectra of jatropha oil (JO), epoxidised jatropha oil (EJO), and jatropha oil-based polyol (JOL).

The FTIR spectra for the jatropha oil (JO), epoxidised jatropha oil (EJO), and jatropha oil‑based polyol (JOL) are shown in Figure 4. From the spectra of the jatropha oil, the absorbance band at 3007 cm^−^^1^ corresponds to the double bond group. The disappearance of the double bond and appearance of new peaks at 840 and 819 cm^−^^1^ in the spectra of the epoxidised jatropha oil confirms the conversion of the double bond to an epoxy group during the epoxidation reaction. After the oxirane ring was opened, the polyol was formed, and this was confirmed by the existence of a broad absorbance band at 3425 cm^−^^1^, which was attributed to the hydroxyl group in the JOL spectra.

From the EJO spectra, the weak and broad shoulder around 3425 cm^−^^1^ attributed to the OH group was observed, instead of a remarkable peak, as reported in other studies [14,15]. This indicates that the minimum side reaction associated with the ring opening of epoxy with water/acid during epoxidation.

### 2.2. Effect of Molar Ratio, Temperature, and Reaction Time on the Oxirane Oxygen Content

**Figure 5:**
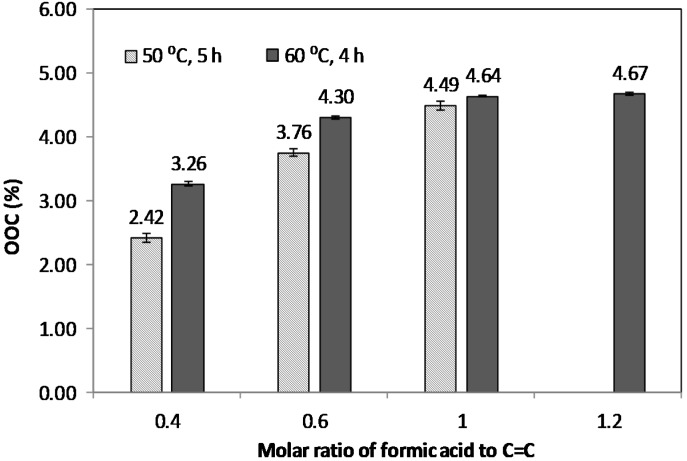
Effect of molar ratio and reaction temperature on the oxirane oxygen content (OOC) of epoxidised jatropha oil.

**Figure 6:**
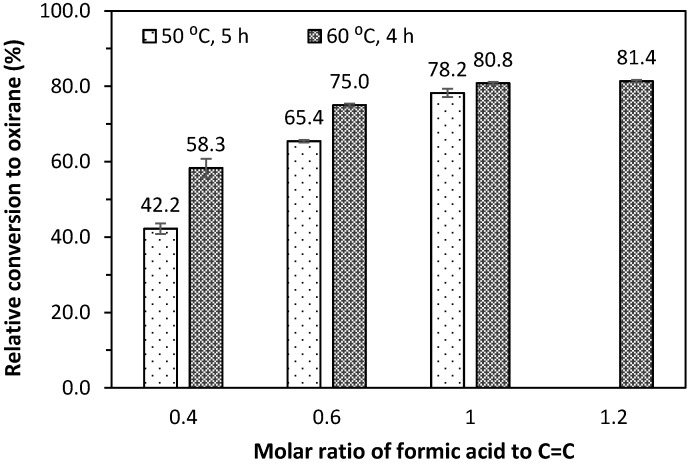
Effect of the molar ratio and reaction temperature on relative conversion to oxirane of epoxidised jatropha oil.

Figure 5 and Figure 6 present the oxirane oxygen content, OOC (%), and relative conversion to oxirane of epoxidised jatropha oil prepared by different molar ratios of formic acid to the oil double bond and reaction temperature. The epoxidation reaction at 50 °C was conducted over 5 h, while the reaction at 60 °C was conducted over 4 h. Overall, increasing the mole ratio of formic acid to the oil double bond from 0.4 to 0.6 increased the OOC from 2.42% to 4.49% and from 3.26% to 4.64% for the reactions conducted at 50 °C and 60 °C, respectively. However, the increase in the OOC becomes less significant when there is a 20% excess of formic acid in the mixture. About 80% relative conversion to oxirane was achieved when the mole of acid and the oil double bond were equal.

The reaction at 60 °C resulted in a better OOC with a reduced reaction time, compared to the reaction conducted at a lower temperature. It was interesting that a 4.3% OOC with a relative conversion to oxirane of 75% could be achieved with a molar ratio of 0.6, a reaction temperature of 60 °C, and a 4 h of reaction time. This was about same value as obtained by Goud and co-workers [12] with the application of an ion exchanger or 2 wt. % sulphuric acid catalyst after 10 h of reaction at 50 °C. The results were comparable with the findings of Hazmi and co-workers [14] for 5 h epoxidation assisted by more concentrated hydrogen peroxide (50%) at a temperature of 65 °C and higher formic acid loading.

A greater concentration of hydrogen peroxide was also employed by Meyer and co-workers [15] at prolonged reaction times of up to 10 h, which resulted in a %OOC of 4.75 at a temperature of 50 °C. These results suggest that a reaction temperature of 60 °C can be employed to achieve a higher conversion to oxirane over a shorter reaction period without the application of a catalyst or more concentrated hydrogen peroxide.

### 2.3. Effect of Oxirane Oxygen Content on OH Numbers of Jatropha Oil-Based Polyol (JOL)

**Figure 7:**
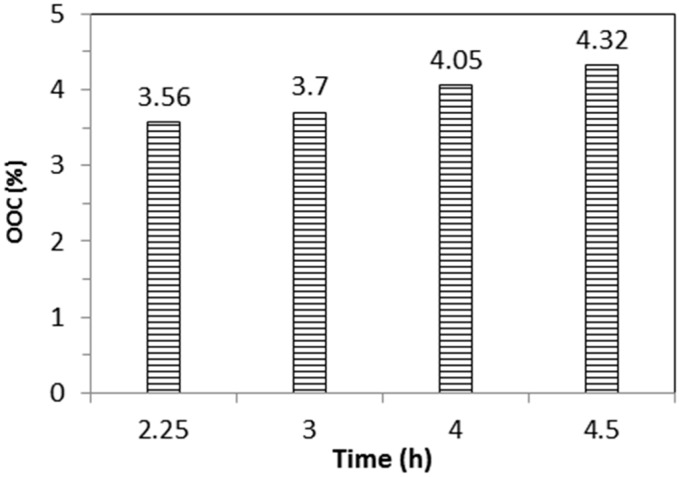
Effect of reaction time on OOC of epoxidised jatropha oil.

A reaction parameter of 60 °C and a molar ratio of 0.6 were selected for the preparation of epoxidised jatropha oil to be further converted to polyol by oxirane ring opening. For this purpose, the amount of JO was scaled up to 400 g. Figure 7 shows the effect of varying reaction times with respect to the OOC. Increasing the reaction time increased the OOC in the epoxidised JO. It can be seen that the OOC obtained at 4 h was lower than the results found in the previous work, in which a small scale sample was used. This is due to the low mass transfer coefficient in a larger scale of preparation, as the other factors such as the heat transfer area are not well considered. At this stage, all the epoxidised JO was converted to polyol by the oxirane ring opening method using methanol.

**Figure 8:**
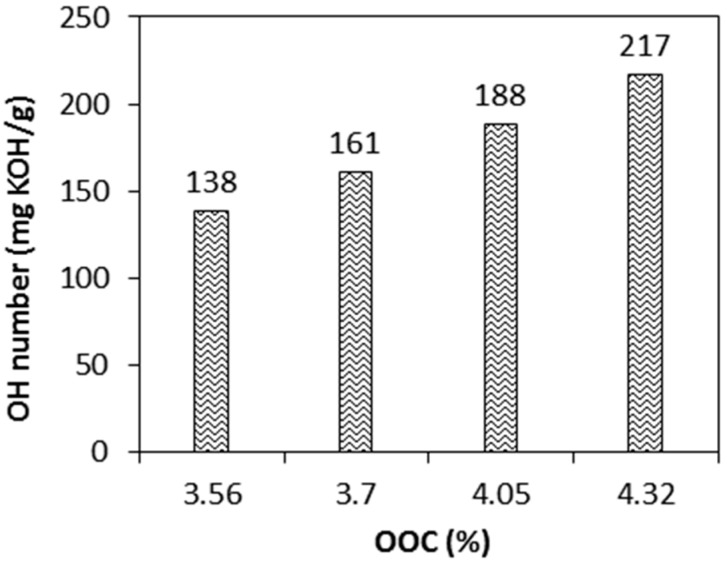
Effect of %OOC of EJO on the OH number of JOL.

The relationship between %OOC and OH number is expressed in Figure 8. As expected, higher OH numbers of polyol were obtained by increasing the %OOC. Polyol with increasing OH numbers from 138–217 mgKOH/g was successfully produced from epoxidised jatropha oil with an OOC increase from 3.56 to 4.32. These values are comparable with other vegetable oil based polyols synthesised from soybean oil, castor oil, linseed oil, and palm oil [16,17,18]. The OH numbers will determine the amount of diisocyanate required for polyurethane synthesis. Depending on the application, vegetable oil-based polyols with relatively high OH numbers are generally desired for production of rigid polyurethane [19]. However, polyols with low OH numbers are advantageous in terms of bio-based content, as they require less diisocyanate as a hard segment in the polyurethane [20]. The combination of the OH numbers and hard segment content was reported as a key role in controlling the structure and the thermophysical and mechanical properties of the polyurethane film [16,17,18,19,20].

### 2.4. Molecular Weight and Rheology of Epoxidised Jatropha Oil (EJO)

**Figure 9:**
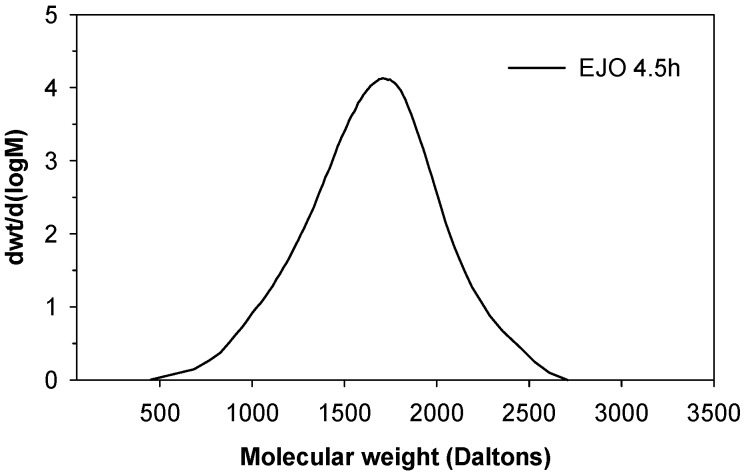
The molecular weight distribution of EJO 4.5 h.

**Figure 10:**
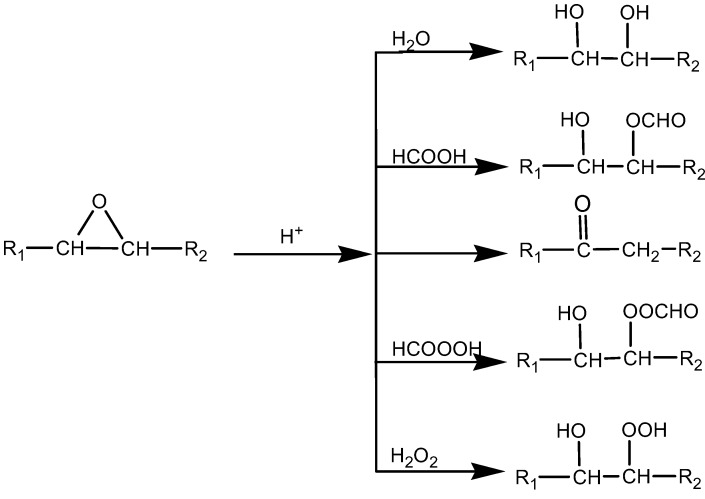
Side reaction of epoxy groups [21].

**Figure 11:**
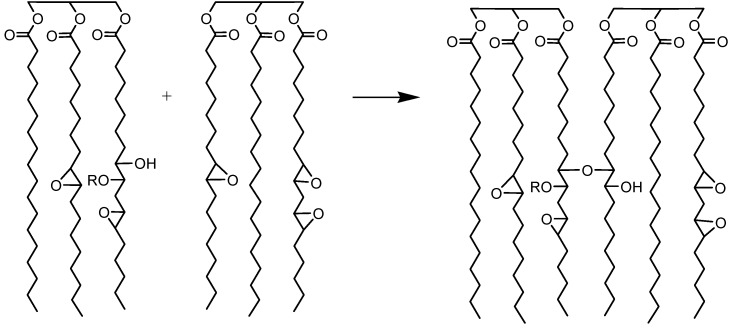
Schematic representation of dimer formation.

The molecular weights of the jatropha oil (JO) and epoxidised jatropha oil (EJO) were determined using gas permeation chromatograpy (GPC). The analysed GPC results (Table 2) for JO and the three EJOs produced at various reaction times, 3 h, 4 h, and 4.5 h, are presented as number average (*M*_n_) and weight average (*M*_w_) molecular weight. A higher polydispersity index (PDI) was observed for EJO 3 h and EJO 4 h, indicating a broad molecular weight distribution, which is a typical characteristic of jatropha oil (JO). The EJO 4.5 produced using a longer reaction time showed a significantly higher *M*_n_ of 1457 with a narrow molecular weight distribution (Figure 9), suggesting the formation of a dimer. Dimerisation (or oligomerisation) is an epoxy ring opening side reaction that occurs when the epoxy group reacts with the acid or water, followed by the dimerisation of the already formed hydroxyl compound. In the case at issue the first step, the attack of a proton on an epoxy group leads to the formation of hydroxyl and acetyl or formyl groups. If the reaction stops at that stage, a polyol is formed (Figure 10) [21]. A hydroxyl group may react further with an epoxy group to give internal ethers or oligomeric ethers (dimers) [21]. The schematic of dimer formation of epoxidised jatropha oil is shown in Figure 11. In addition to increasing molecular weight, dimerisation typically will cause a significant increase of viscosity[21].

**Figure 12:**
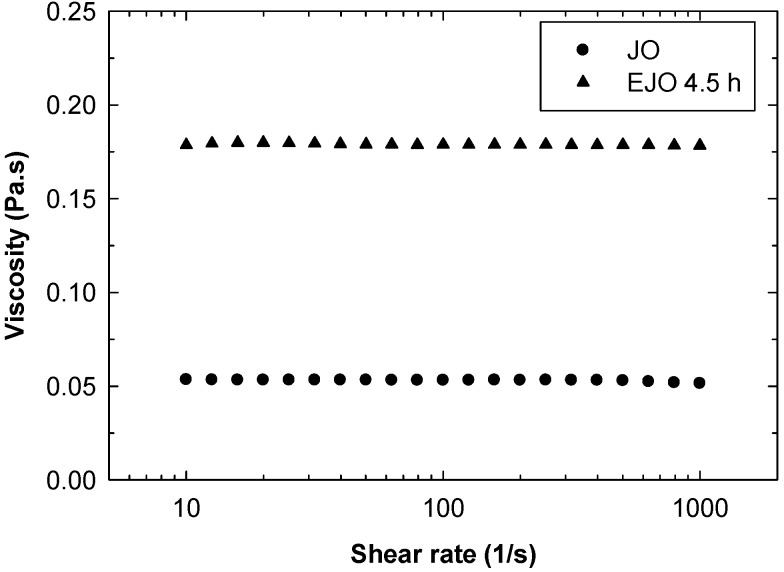
Viscosity curve of jatropha oil (JO) and epoxidised jatropha oil (EJO) at 25 °C.

**Figure 13:**
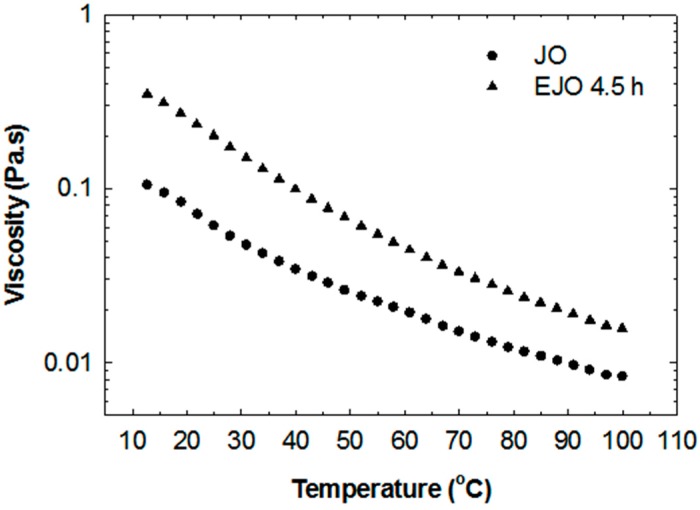
Viscosity of jatropha oil (JO) and epoxidised jatropha oil (EJO) at various temperatures.

The functionalisation of double bonds to the epoxy groups leads to a significant increase in viscosity. As shown in Figure 12, the viscosity of EJO 4.5 h at 25 °C is 180 mPas, while the viscosity of the starting jatropha oil is 55 mPas. Both JO and EJO 4.5 h exhibit Newtonian behaviour in the measured shear rate ranges. Increasing the temperature causes a significant reduction of viscosity, as shown in Figure 13. Although having a dimer structure, the viscosity of the EJO 4.5 h is still low and comparable to completely epoxidised soybean oil (ESO), as reported by Petrovic and co-workers [21]. It is worth noting that a low viscosity ESO produced commercially has been receiving attention for use as a reactive diluent to reduce the viscosity of a petroleum-based epoxy, thus improving the processibility of the resin [22].

### 2.5. Molecular Weight, Physicochemical and Rheological Properties of Jatropha Oil-based Polyol (JOL)

The molecular weight of JOL is presented in Table 3. It is obvious that the molecular weight is higher than the starting EJO. With *M*_n_ increased from a range of 834–1457 for EJO to 1439–2129 for JOL, it is believed that oligomerisation also occurred during the ring opening step with methanol. The newly formed OH groups tend to react with the existing epoxy groups to form a higher molecular weight dimer or oligomer. Oligomerisation is more pronounced in JOL 217 as a result of the relatively high epoxy content of the starting EJO. The formation of oligomers was also reported by Caillol and co-workers [23] for soybean oil-based polyol (44%–63% oligomers) produced by ring opening with carboxylic acid.

Other physico-chemical properties of the JOL are given in Table 4. The polyols produced were slightly yellow in colour, and all of them were liquid at room temperature. A polyol of industrial importance is commonly required to have low viscosity and a high hydroxyl value. From our previous work, waterborne polyurethane film synthesised from JOL 138 was soft and tacky with poor structural rigidity [20]. For this reason, only JOL 161, JOL 188, and JOL 217 were analysed in terms of viscosity and rheology. In polyurethane production, viscosity is an important criterion as it determines the ease of pouring and pumping the polyol into the machine to be mixed with diisocyanates [18]. As shown in Figure 10, the viscosity of the JOL increased from 430 to 940 mPas when the OH number increased from 161 to 217 mgKOH/g. The viscosity is comparable with soybean oil-based polyol [24,25]. With a lower OH number, a viscosity of 2500 to 35,000 mPas was reported for polyol derived from crude palm oil [18]. A commercial polyol is available in various viscosity ranges from as low as 230 mPas up to above 10,000 mPas, depending on the OH number, functionality, production routes, and its vegetable oil origin types [26].

**Figure 14:**
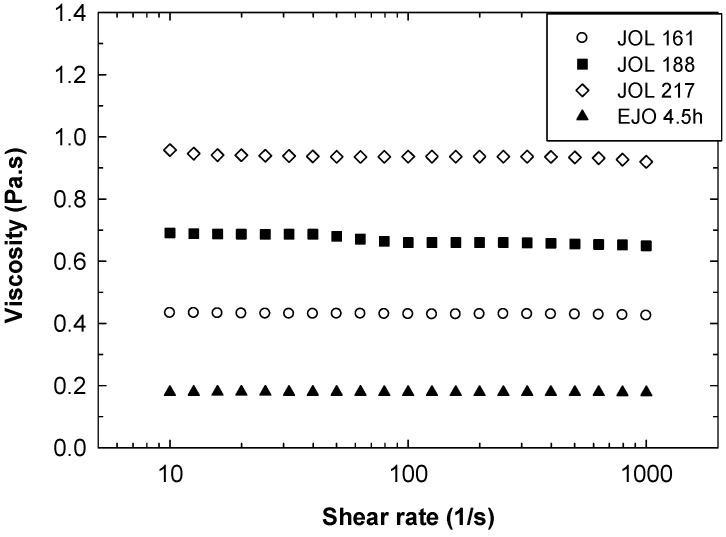
Viscosity curves of jatropha oil-based polyol with different OH numbers at 25 °C.

**Figure 15:**
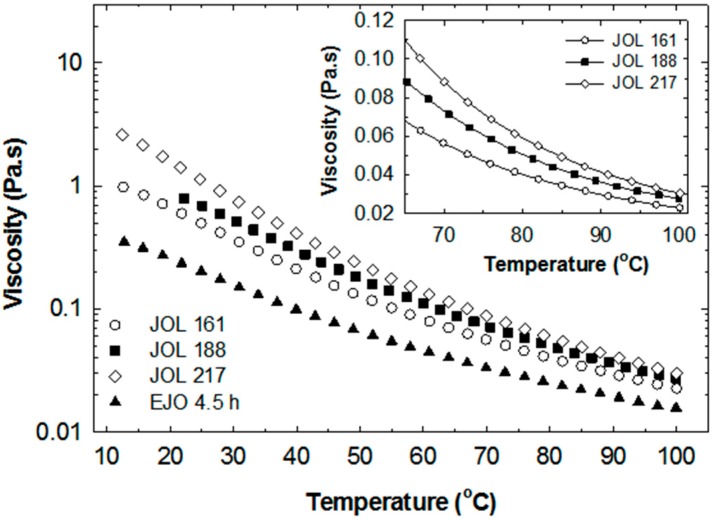
Viscosity of jatropha oil-based polyol (JOL) at various temperatures at a shear rate of 250 s^−^^1^.

The JOL exhibits Newtonian behaviour similar to the rheological behaviour of the starting EJO, as shown in Figure 14. With increasing temperature, the viscosity decreased significantly (Figure 15). Based on these results, the processing temperature of the JOL could be easily correlated with viscosity. For example, the JOL has to be heated to a temperature above 70 °C to achieve a viscosity of less than 100 mPas (see inset in Figure 15). In waterborne polyurethane production, the reaction temperature is usually in the range of 65 °C to 90 °C [16,27,28].

**Figure 16:**
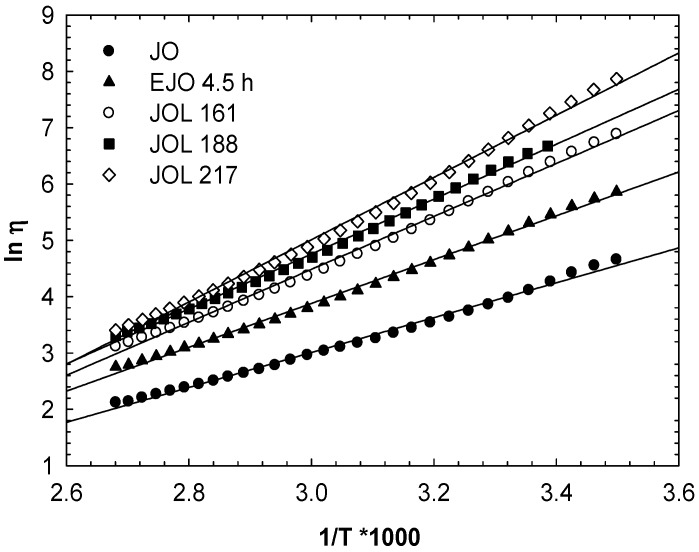
The viscosity–temperature relationship for jatropha oil-based polyols (JOLs).

As shown in Figure 16, the viscosity plotted against the reciprocal temperature obeyed the Arrhenius dependence (Equation (1)):

(1) $$\eta =\eta_{o}e^{\frac{E}{RT}}$$

where *η_o_* is a reference viscosity, *E* is the viscous-flow activation energy, *R* is the gas constant, and *T* is the absolute temperature (K). From the slopes of these straight lines, the activation energy of flow can be calculated, and this is presented in Table 5.

The viscous-flow activation energies for jatropha oil and EJO are also included. The activation energy of flow is related to the flexibility of the molecular chains and the interaction between molecules [29]. The activation energies of the polyols were significantly higher than those of EJO and jatropha oil. These confirmed the hydrogen bonding present in all the JOLs, increasing according to hydroxyl numbers. The activation energy values obtained are comparable to those values of soy polyols reported by Dai and co-workers [25]. Vegetable oil contains a *cis* C=C double bond that created a defect in the packing of the triglycerides. The lower viscosity of the vegetable oil is comparable to animal fat [30]. The activation energy of jatropha oil is similar to that reported for soybean oil [29].

### 2.6. Monitoring Polyurethane Synthesis by FTIR

**Figure 17:**
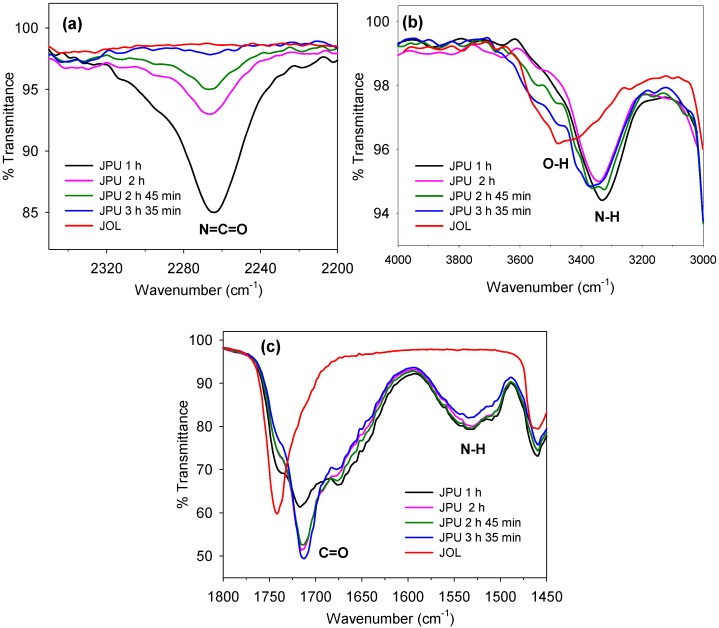
FTIR spectra of (**a**) the isocyanate group in polyurethane, (**b**) the hydroxyl and urethane group in polyol and polyurethane respectively, and (**c**) the carbonyl and urethane group in polyurethane.

The polyurethane synthesis was monitored by FTIR analysis, as shown in Figure 17. At 1 h of reaction time, a strong absorbance peak at 2270 cm^−^^1^ was observed, which indicated a high amount of free isocyanate. The formation of an absorbance peak at 3324 cm^−^^1^ confirmed the formation of urethane by the reaction of dimethylol propionic acid (DMPA) and polyol with isophrene diisocyanate (IPDI). At the early stage of polymerisation, it is believed that the DMPA reacted rapidly with IPDI due to the high reactivity of the primary hydroxyl in the structure.

The consumption rate of free isocyanate is high during the first two hours of reaction and becomes slower as time proceeds due to a decreasing amount of NCO groups and the low reactivity of secondary OH groups in the JOL. After 3 h of reaction, hydroxyethyl methacrylate (HEMA) was added to end-cap the terminal NCO groups. After 3 h and 35 min, the NCO peak almost disappeared, thus indicating that most of the isocyanate groups were involved in polymerisation. An absorbance peak at 1707 cm^−^^1^ corresponded with carbonyl group stretching, and the –NH bending observed at 1531 cm^−^^1^ was evidence of polyurethane formation.

### 2.7. Physical Properties of the Aqueous Polyurethane (JPU) Dispersion

**Figure 18:**
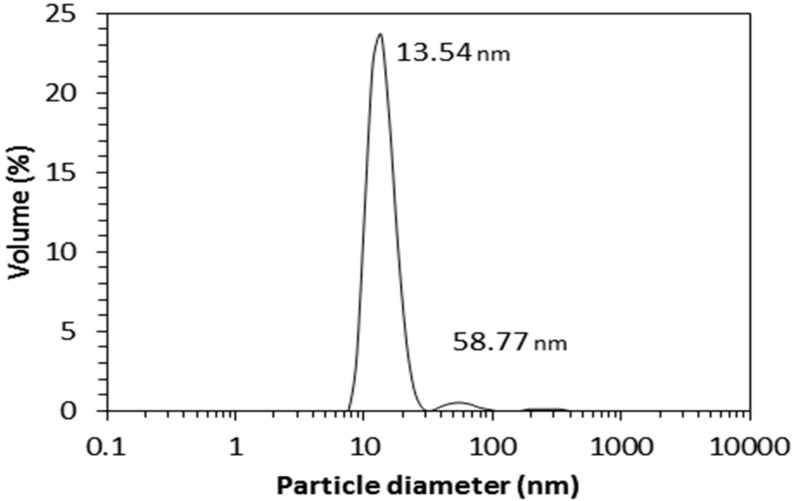
Particle size distribution of the JPU dispersion.

The aqueous JPU dispersion synthesised in this work has a solid content of 25 wt. % and appears milky white in colour. The dispersion has a low viscosity of 12.5 mPas, with a zeta potential of −56.6 mV. Figure 18 shows a particle size distribution of the dispersion, which demonstrates the low particle size with a modal peak at 13.5 nm. From the point of view of colloidal stability, the dispersion is stable as the average particle size is less than 100 nm [31]. The particle size is an important parameter in deciding the industrial applications of a waterborne polyurethane dispersion [32], in which a small particle size is essential for deep penetration of the dispersion into a substrate [33].

## 3. Materials and Methods

### 3.1. Materials

Reagent grade hydrogen peroxide 30% and methanol were supplied by Merck (Darmstadt, Germany). Isophrene diisocyanate (IPDI), dimethylol propionic acid (DMPA), *n*-methyl pyrollidone (NMP), hydroxyethyl methacrylate (HEMA), phtalic anhydride, and dibutyltin dilaurate (DBTDL) were supplied by Sigma Aldrich (Milwaukee, WI, USA). Ethyl methyl ketone (MEK), triethylamine (TEA), formic acid, magnesium sulphate anhydrous, pyridine, and sodium hydroxide were reagent grade, supplied by Classic Chemicals (Shah Alam, Malaysia). The crude jatropha oil was supplied by Biofuel Bionas Sdn Bhd, Kuala Lumpur, Malaysia. It is a non-food grade material and was used as received. All chemicals were used without further purification.

### 3.2. Preparation of Jatropha Oil-Based Polyol (JOL)

Polyols were synthesised by a two-step process; namely epoxidation followed by oxirane ring opening (Figure 2).

#### 3.2.1. Epoxidation

The reaction parameters for the epoxidation step are presented in Table 6. The epoxidation parameters are divided into three groups; reaction temperature at 50 °C with varied molar ratio, reaction at 60 °C with varied molar ratio, and the molar ratio fixed in 1:0.6:1.7 with varied reaction times. Briefly, a mixture of jatropha oil and formic acid was placed in a 1 L flask and heated to 40 °C under continuous stirring. Hydrogen peroxide 30% was then added dropwise for 30 min before increasing the temperature to a specified reaction temperature. After a prescribed time, the mixture was cooled to room temperature. The aqueous layer was discarded, and the remaining acid was removed by washing the oil layer with an excessive amount of water. Magnesium sulphate was then added to the EJO as a drying agent.

#### 3.2.2. Oxirane Ring Opening

The reactions were carried out in a four-necked flask equipped with a mechanical stirrer, condenser, dropping funnel, and thermometer. For this reaction, the molar ratio of methanol to water is 5:1, while the amount of water is 10% (*w*/*w*) of epoxidised jatropha oil. Briefly, a calculated amount of methanol, water, and sulphuric acid catalyst (0.3 wt. %) were poured into a 1 L flask and heated to 64 °C under continuous stirring. Epoxidised jatropha oil was then added, and the reactions proceeded for 30 min, followed by adding sodium bicarbonate to quench the reaction. After being cooled to room temperature, the deposit was discarded. Methanol and water were removed by vacuum distillation. Only the third series of epoxidised jatropha oil was selected to be further converted to polyol using the same oxirane ring opening conditions. Four types of polyol with different hydroxyl numbers were produced.

### 3.3. Preparation of Aqueous Polyurethane Dispersion

**Figure 19:**
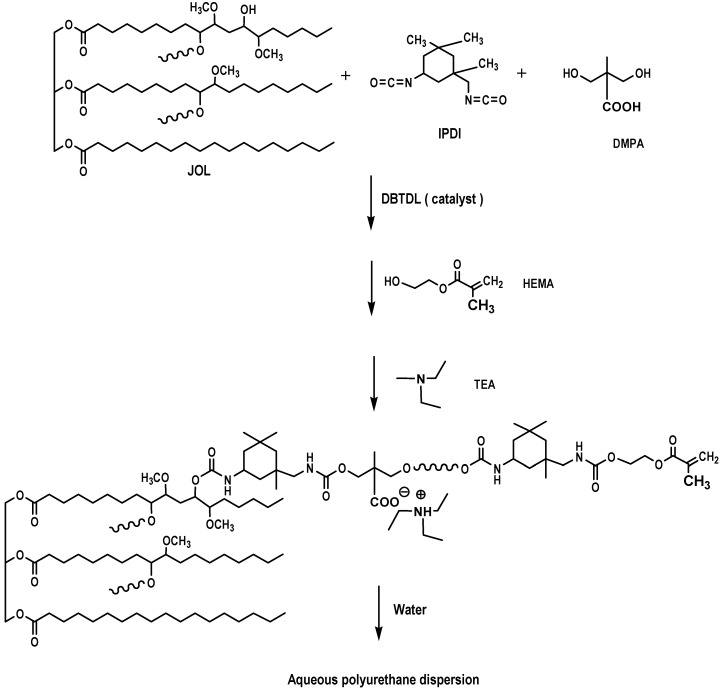
Synthesis of aqueous polyurethane dispersion.

The reactions were carried out in a four-necked flask equipped with a mechanical stirrer, dropping funnel, condenser, and thermometer. The jatropha oil-based polyol (JOL 161) and DMPA (dissolved in NMP) were charged into the flask and heated to a temperature of 80 °C for 30 min. The mixture was then cooled to 60 °C and the IPDI was slowly dropped for 30 min. DBTDL was used as a catalyst. The reaction was reheated and reached a temperature of 80 °C for 3 h, followed by the addition of HEMA for a chain termination step. About 20 g of MEK was added batch by batch to reduce the viscosity of the system (the total amount of MEK was 100 g). The reaction was complete when the ATR-FTIR spectra showed that the NCO peak at 2270 cm^−^^1^ had disappeared, thus indicating that all the diisocyanate was consumed. The reactants were then cooled to 40 °C and neutralised by the addition of TEA (1.2 equiv. per DMPA), followed by dispersion at high speed with distilled water to produce the JPU dispersions with a solid content of ∼25 wt.% after removal of the MEK under vacuum. The formulation of waterborne JPU is shown in Table 7, using NCO/OH of 0.8/1.0, while the chemical reaction is shown in Figure 19.

### 3.4. Characterisation

The oxirane oxygen content measurement of the epoxidised jatropha oil was determined according to AOCS Tentative Method Cd 9-57, revised in 1963. The theoretical maximum oxirane oxygen content, $OOC_{t}$ was determined to be 5.74% according to Equation (2):

(2) $$\;\;\;OOC_{t}=\left\{\left(IV_{0}/2A_{i}\right)/\left[100+\left(IV_{0}/2A_{i})A_{0}\right)\right]\right\}\;\times A_{0\;}\times 100$$

where $IV_{0}$ (96.06) is the initial iodine value of the oil, $A_{i}$ (126.9) is the atomic weight of iodine, and $A_{0}$ (16.0) is the atomic weight of oxygen [13].

The oxygen oxirane content was used to determine the relative conversion to oxirane, according to Equation (3):

(3) $$\;Relative\;conversion\;to\;oxirane=OOC_{c}/OOC_{t}\;\;$$

where *OOC_c_* is an experimental oxirane oxygen content.

The determination of the hydroxyl number of the jatropha-based polyol followed ASTM D4274-99 Test Method C (reflux phthalation) standard practice. The equivalent weights (EW) of the polyols were calculated using Equation (4) [18]:

(4) $$\mathrm{EW}=\frac{\left(\mathrm{Molecular}\;\mathrm{weight}\;\mathrm{of}\;\mathrm{KOH}\;\times \;1000\right)}{\mathrm{OH}\;\mathrm{number}}\;\;=\frac{56100}{\mathrm{OH}\;\mathrm{number}}\;\;\;\;\;\;\;\;\;\;\;\;\;\;\;\;\;\;\;\;\;\;\;\;\;\;\;\;\;\;\;\;\;\;\;\;\;\;\;$$

The acid number measurements of the epoxidised jatropha oil and the polyol were determined according to ASTM D4662-03 Test Method A standard practice.

The FTIR spectra were recorded on a Perkin-Elmer Spectrum 2000 spectrometer (Perkin Elmer, Norwalk, CT, USA), equipped with a horizontal germanium attenuated total reflectance (ATR) accessory. The spectra were recorded in the range of 4000–500 cm^−^^1^ with a nominal resolution of 4 cm^−^^1^.

The molecular weight of the samples was determined using Gel Permeation Chromatography (Waters Co., Milford, MA, USA) with a series of Styragel^®^ columns. Calibration was performed using a polystyrene standard, whereby tetrahydrofuran (THF) was used as the eluent with a flow rate of 1.0 µL/min at 35 °C. The sample injection volume was 50.0 µL.

The rheological properties of the samples were analysed on an ARG2 rheometer (TA Instruments, DE, USA). The rheometer was equipped with a 40 mm 2° cone, and the gap between the truncated tip of the cone and the bottom Peltier plate was maintained at 56 µm.

The particle size of the polyurethane dispersions was measured by a Zetasizer Nano-S (Malvern Instruments, Herrenberg, Germany). Approximately 0.1 mL of the polyurethane dispersion was diluted with 3 mL distilled water and measured at 25 °C.

## 4. Conclusions

Jatropha oil-based polyols were successfully synthesised by epoxidation and an oxirane ring opening method, and the physicochemical properties of the products were reported. The epoxidation reaction parameters such as temperature, the oil double bond to formic acid molar ratio, and time showed a significant effect on the conversion of the double bond to an oxirane group. About 75% conversion was obtained at a temperature of 60 °C, a molar ratio of 1:0.6, and a 4 h reaction time, which is equivalent to 4.30% OOC of epoxidised jatropha oil (EJO). Furthermore, a series of JOLs with a hydroxyl number of 138–217 mgKOH/g was successfully prepared after the oxirane ring opening of the EJO produced at a reaction time of 2.25 to 4.5 h. The resulting JOLs exhibited Newtonian behaviour, with a low viscosity of 430–970 mPas, and molecular weight of 1349–2129 g/mol. Polyurethane was successfully synthesised from JOL, as monitored by FTIR. The corresponding polyurethane dispersion had average small particle size, indicating good colloidal stability.

## Figures and Tables

**Table 1:** List of fatty acid compositions in jatropha oil [9].

Fatty Acid	Unsaturates			Saturates	
	Palmitoleic (C_16/1_)	Oleic (C_18/_)	Linoleic (C_18/2_)	Palmitic (C_16/0_)	Stearic (C_18/0_)
%	1.4	43.1	34.4	14.2	6.9
Total (%)	78.9			21.1	

**Table 2:** Molecular weight of epoxidised jatropha oil.

Sample	*M*_n_	*M*_w_	PDI = *M*_w_/*M*_n_
JO	1083	1630	1.51
EJO 3 h	995	1666	1.67
EJO 4 h	834	1516	1.82
EJO 4.5 h	1457	1566	1.07

**Table 3:** Molecular weight of JOL.

Sample	*M*_n_	*M*_w_	PDI = *M*_w_/*M*_n_
JOL 161	1572	2121	1.35
JOL 188	1439	2110	1.47
JOL 217	2129	2231	1.05

**Table 4:** Physicochemical properties of the JOL.

Sample	OH Number (mgKOH/g)	Acid Value (mgKOH/g)	EW *	f **	Density (kg/m^3^)	Physical Appearance
JOL 138	138	29.92	407	-	-	liquid
JOL 161	161	26.09	348	4.51	965.5	liquid
JOL 188	188	26.66	298	4.82	971.9	liquid
JOL 217	217	15.95	259	8.24	975.5	liquid

* Equivalent weight, ** functionality.

**Table 5:** Viscosity at 45 °C and activation energy of viscous flow (E_a_) for the jatropha oil-based polyols.

Sample	Viscosity at 45 °C (mPas)	Activation Energy (kJ/mol)
JO	28.7	25.7
EJO	77.2	32.3
JOL 161	154.8	39.1
JOL 188	241.0	40.5
JOL 217	289.0	46.0

**Table 6:** Reaction parameters for the epoxidation step and the acid values of epoxidised jatropha oil (EJO).

No.	Molar Ratio ^a^	Reaction Temperature (°C)	Reaction Time (h)	Acid Value (mgKOH/g)
1	1.0:0.4:1.7	50	5	14.49
2	1.0:0.6:1.7	50	5	14.76
3	1.0:1.0:1.7	50	5	16.27
4	1.0:0.4:1.7	60	4	15.10
5	1.0:0.6:1.7	60	4	15.20
6	1.0:1.0:1.7	60	4	16.40
7	1.0:1.2:1.7	60	4	14.43
8	1.0:0.6:1.7	60	2	-
9	1.0:0.6:1.7	60	3	-
10	1.0:0.6:1.7	60	4	-
11	1.0:0.6:1.7	60	4.5	15.95

^a^ Double bond: Formic acid: Hydrogen peroxide.

**Table 7:** The recipe of aqueous polyurethane (JPU) dispersions.

Ingredient	Functional Group	Mass (g)	Mol
JOL	OH	60.0	0.172
DMPA	OH	7.3	0.111
HEMA	OH	4.3	0.033
IPDI	NCO	29.0	0.260
